# Large Variations in the Prices of Urologic Procedures at Academic Medical Centers 1 Year After Implementation of the Price Transparency Final Rule

**DOI:** 10.1001/jamanetworkopen.2022.49581

**Published:** 2023-01-05

**Authors:** Zeynep G. Gul, Danielle R. Sharbaugh, Cailey J. Guercio, Daniel L. Pelzman, Cameron A. Jones, Emily C. Hacker, Vivian I. Anyaeche, Levi Bowers, Ashti M. Shah, Michael G. Stencel, Jonathan G. Yabes, Bruce L. Jacobs, Benjamin J. Davies

**Affiliations:** 1 Division of Urology, University of Washington in St Louis, St Louis, Missouri; 2 Department of Urology, University of Pittsburgh Medical Center, Pittsburgh, Pennsylvania; 3 University of Pittsburgh School of Medicine, Pittsburgh, Pennsylvania; 4 Department of Medicine, University of Pittsburgh Medical Center, Pittsburgh, Pennsylvania

## Abstract

**Importance:**

Patients with urologic diseases often experience financial toxicity, defined as high levels of financial burden and concern, after receiving care. The Price Transparency Final Rule, which requires hospitals to disclose both the commercial and cash prices for at least 300 services, was implemented to facilitate price shopping, decrease price dispersion, and lower health care costs.

**Objective:**

To evaluate compliance with the Price Transparency Final Rule and to quantify variations in the price of urologic procedures among academic hospitals and by insurance class.

**Design, Setting, and Participants:**

This was a cross-sectional study that determined the prices of 5 common urologic procedures among academic medical centers and by insurance class. Prices were obtained from the Turquoise Health Database on March 24, 2022. Academic hospitals were identified from the Association of American Medical Colleges website. The 5 most common urologic procedures were cystourethroscopy, prostate biopsy, laparoscopic radical prostatectomy, transurethral resection of the prostate, and ureteroscopy with laser lithotripsy. Using the corresponding *Current Procedural Terminology* codes, the Turquoise Health Database was queried to identify the cash price, Medicare price, Medicaid price, and commercial insurance price for these procedures.

**Exposures:**

The Price Transparency Final Rule, which went into effect January 1, 2021.

**Main Outcomes and Measures:**

Variability in procedure price among academic medical centers and by insurance class (Medicare, Medicaid, commercial, and cash price).

**Results:**

Of 153 hospitals, only 20 (13%) listed a commercial price for all 5 procedures. The commercial price was reported most often for cystourethroscopy (86 hospitals [56%]) and least often for laparoscopic radical prostatectomy (45 hospitals [29%]). The cash price was lower than the Medicare, Medicaid, and commercial price at 24 hospitals (16%). Prices varied substantially across hospitals for all 5 procedures. There were significant variations in the prices of cystoscopy (χ^2^_3_ = 85.9; *P* = .001), prostate biopsy (χ^2^_3_ = 64.6; *P* = .001), prostatectomy (χ^2^_3_ = 24.4; *P* = .001), transurethral resection of the prostate (χ^2^_3_ = 51.3; *P* = .001), and ureteroscopy with laser lithotripsy (χ^2^_3_ = 63.0; *P* = .001) by insurance type.

**Conclusions and Relevance:**

These findings suggest that, more than 1 year after the implementation of the Price Transparency Final Rule, there are still large variations in the prices of urologic procedures among academic hospitals and by insurance class. Currently, in certain situations, health care costs could be reduced if patients paid out of pocket. The Centers for Medicare & Medicaid Services may improve price transparency by better enforcing penalties for noncompliance, increasing penalties, and ensuring that hospitals report prices in a way that is easy for patients to access and understand.

## Key Points

**Question:**

Are academic medical centers compliant with the Price Transparency Final Rule, and how does the price of urologic procedures vary among hospitals and by insurance class ?

**Findings:**

In this cross-sectional analysis of 153 academic hospitals, compliance with the mandate was low, and there were large variations in the price of procedures among hospitals. There were also significant differences in the price of 5 urologic procedures by insurance class (Medicare, Medicaid, commercial insurance, and cash price), with the cash price being the lowest reported at 16% of hospitals.

**Meaning:**

These findings suggest that more than 1 year after the implementation of the Price Transparency Final Rule, there are still large variations in the prices of urologic procedures among academic hospitals and by insurance class.

## Teaser

This cross-sectional study examines compliance with the Price Transparency Final Rule and quantifies variations in the price of urologic procedures among academic hospitals and by insurance class.

## Introduction

The US spends almost twice as much on health care as other developed nations, in part because of the higher prices US individuals pay for health care products and services.^1^ For the 31.6 million US individuals without health insurance and the 40% of privately insured US individuals with high-deductible health plans, these high prices can translate to high out-of-pocket costs.^2,3,4^ High out-of-pocket costs represent a substantial financial burden for some patients, who may then forgo or delay necessary care.^5,6^

To improve patient care and combat rising prices, the Centers for Medicare & Medicaid Services (CMS) passed the Hospital Price Transparency Final Rule, which requires that hospitals publish their standard charges in a publicly available, machine-readable file.^7^ Price transparency is a prerequisite for price shopping, and according to standard economic theory, price shopping ensures that identical goods at different locations have the same price (the Law of One Price). Theoretically, the Hospital Price Transparency Final Rule would make it possible for patients to price shop, which should encourage competition and lead to lower and more uniform prices.^7^

Approximately 1 in 4 patients with a urologic disease experiences financial toxicity, which are defined as the financial burden and stress associated with receiving care.^8,9,10^ High levels of financial toxicity are associated with care delays and poor health care–related quality of life. For these patients, price transparency, and the subsequent reductions in cost, could greatly improve the affordability of care and reduce financial toxicity.^11,12^ To better understand the impact of price transparency on urologic care, we examined prices for 5 common urologic procedures among academic medical centers. We also compared prices among different insurances classes, including commercial, Medicaid, Medicare, and the cash price.

## Methods

### Hospitals and Procedures

This cross-sectional study followed the Strengthening the Reporting of Observational Studies in Epidemiology (STROBE) reporting guidelines. This study did not involve any human participants and so did not require institutional review board approval or informed consent, in accordance with 45 CFR §46. Using the Association of American Medical Colleges website, we identified all academic hospitals. Exclusion criteria included duplicate hospitals (identified by the same address or if 1 address corresponded to a corporate office building), health care systems (with multiple hospitals that were not independently listed on the website), specialty hospitals that do not provided urologic care, children’s hospitals, and Veterans Affairs hospitals (which are not required to disclose prices).

Using data from our multihospital, academic institution, we identified the 5 most common urological procedures performed during 2020 and 2021. These were cystourethroscopy, prostate biopsy, laparoscopic radical prostatectomy, transurethral resection of the prostate (TURP), and ureteroscopy with laser lithotripsy (URS/LL). We used the *Current Procedural Terminology* (*CPT*) codes for these procedures (52000, 55700, 55866, 52601, and 52353, respectively) to determine the associated costs. To ensure that case volumes at our institution were representative of those at other academic medical centers, we identified the most common procedures performed by urology residents according to Accreditation Council for Graduate Medical Education case logs. As described by Silvestre et al,^13^ from 2010 to 2018, the most commonly logged procedures were transurethral surgery (which would include both cystoscopy and TURP as well as others), ureteroscopy (with or without laser lithotripsy), prostate biopsy, and radical prostatectomy (with or without laparoscopic assistance).

### Data Source

We obtained all pricing information from Turquoise Health, a data service company that compiles pricing information from the machine-readable files hospitals must provide to comply with CMS regulations.^14^ Hospitals are required to disclose 5 standard charges, including (1) the gross prices, (2) the payer-specific negotiated prices, (3) the discounted cash prices, (4) the deidentified minimum negotiated prices, and (5) the deidentified maximum negotiated prices for at least 300 shoppable services. The gross price is the price as listed on the hospital chargemaster before any negotiations with third-party payers (which would then be the payer-specific negotiated charges), and the discounted cash price is the price that would apply to self-pay or uninsured patients. Shoppable services are defined as “a service package that can be scheduled by a healthcare consumer in advance.”^7^ CMS has identified 70 required shoppable services, and the remaining 230 are chosen by the hospital.^7^ Of the 5 procedures included in our analysis, only prostate biopsy (*CPT* code 55700) and laparoscopic radical prostatectomy (*CPT* code 55866) are included in the CMS-specified list of shoppable services

The Turquoise Health Database has been used for several recent analyses.^15,16,17^ Using *CPT* codes, we queried the database for procedure costs as of March 24, 2022. We included costs for the following payer classes: commercial, Medicaid, Medicare, and the cash price. The Medicaid price was generated by Turquoise Health from Medicare’s outpatient prospective payment system and accounts for both the hospitals’ location and the wage index. Although hospitals are not required to disclose Medicare or Medicaid prices, we included these in our analysis to help contextualize commercial and discounted cash prices. Among commercial payers, we included Aetna, Blue Cross/Blue Shield, Cigna, and United Healthcare. If our query did not yield a unique cost in the Turquoise Health Database, we referenced the hospital’s chargemaster to determine which price to include. If the chargemaster was uninformative, we used the median price for analysis. The median commercial insurance price was defined as the median price of all the plans listed for each commercial insurer.

When provided by the hospitals, the database included details on where the procedure was performed (eg, office, operating room, ambulatory care center, or interventional radiology suite), if it was performed inpatient or outpatient, and any associated professional fees. However, these data were not consistently reported for several reasons. First, the CMS mandate only requires hospitals (not ambulatory care centers or clinics) to report prices. Second, hospitals are not required to report professional fees. Third, hospitals have to report both the inpatient and outpatient procedure price only if these prices are different (or if the hospital withholds this information and is not in full compliance).

Because cost was determined by *CPT* code, we excluded all prices that were specifically identified as an inpatient price; outpatient prices and prices that did not have a designation were included. We also excluded costs for services provided in the emergency department. To make our findings more applicable to everyday practice, we included the prices of only procedures performed in clinic or at an unspecified location for cystourethroscopy (*CPT* code 52000) and prostate biopsy (*CPT* code 55700). Similarly, we included prices only for procedures performed in the operating room or at an unspecified location for URS/LL (*CPT* code 52353), TURP (*CPT* code 52601), and laparoscopic radical prostatectomy (*CPT* code 55866).

### Statistical Analysis

First, we determined the percentage of chosen hospitals that reported a procedure price for all 4 insurance classes and for all 4 commercial insurance types. Next, we determined how often the cash price was the lowest price reported for each of the 5 procedures among the hospitals. For each procedure, we compared prices among both the 4 insurance classes and among the 4 commercial insurance types using Kruskal-Wallis tests. We created violin plots to illustrate variations in the price among the different insurance classes for each procedure. We calculated the medians and IQRs of prices among insurance classes and commercial insurance types, to examine how the prices varied among reporting hospitals. As a sensitivity analysis, we compared both the highest and lowest negotiated prices by insurance class and by commercial insurance type. Analysis was performed with SAS statistical software version 9.4 (SAS Institute, Inc) and R statistical software version 4.1.0 (R Project for Statistical Computing). All tests were 2-sided and the probability of a type I error was set at α = .05.

## Results

Among the 153 academic hospitals, commercial prices were reported more often for cystourethroscopy (86 hospitals [56%]) and prostate biopsy (81 hospitals [53%]) than for URS/LL (46 hospitals [30%]), TURP (47 hospitals [31%]), and laparoscopic radical prostatectomy (45 hospitals [29%]). The commercial prices were reported more often than the cash price (Table 1). Because laparoscopic prostatectomy is on the list of 70 shoppable services, just 29% of hospitals were compliant with the price transparency legislation. Compared with the other insurance classes, the cash price was the lowest reported price among 24 of 152 hospitals (16%) that reported prostate biopsy charges, 9 of 74 hospitals (12%) that reported laparoscopic prostatectomy, 17 of 141 hospitals (12%) that reported cystourethroscopy charges, 7 of 71 hospitals (10%) that reported URS/LL charges, and 5 of 67 hospitals (7%) that reported TURP charges (Table 2).

**Table 1.** Hospitals With Price Listed for Each Procedure by Insurance Type

Procedure and insurance type	Hospitals, No. (%) (N = 153)
Cystourethroscopy	
Commercial	86 (56)
Medicaid	51 (33)
Medicare reference pricing	131 (86)
Cash	70 (46)
Prostate biopsy	
Commercial	81 (53)
Medicaid	45 (29)
Medicare reference pricing	141 (92)
Cash	71 (46)
Prostatectomy	
Commercial	45 (29)
Medicaid	25 (16)
Medicare reference pricing	68 (44)
Cash	31 (20)
Transurethral resection of the prostate	
Commercial	47 (31)
Medicaid	27 (18)
Medicare reference pricing	60 (39)
Cash	24 (16)
Ureteroscopy with laser lithotripsy	
Commercial	46 (30)
Medicaid	32 (21)
Medicare reference pricing	65 (42)
Cash	30 (20)

**Table 2.** Hospitals With the Lowest Price by Insurance Type for Each Procedure

Procedure and insurance type	Hospitals, No. (%)^a^
Cystourethroscopy (n = 141)	
Commercial	4 (3)
Medicaid	11 (8)
Medicare reference pricing	109 (77)
Cash	17 (12)
Prostate biopsy (n = 152)	
Commercial	6 (4)
Medicaid	29 (19)
Medicare reference pricing	93 (61)
Cash	24 (16)
Prostatectomy (n = 74)	
Commercial	5 (7)
Medicaid	22 (30)
Medicare reference pricing	38 (51)
Cash	9 (12)
Transurethral resection of the prostate (n = 67)	
Commercial	1 (2)
Medicaid	27 (40)
Medicare reference pricing	35 (51)
Cash	5 (7)
Ureteroscopy with laser lithotripsy (n = 71)	
Commercial	3 (4)
Medicaid	30 (42)
Medicare reference pricing	31 (44)
Cash	7 (10)

^a^ To be included, each hospital had to list at least 3 of 4 insurance types.

**Figure 1.**
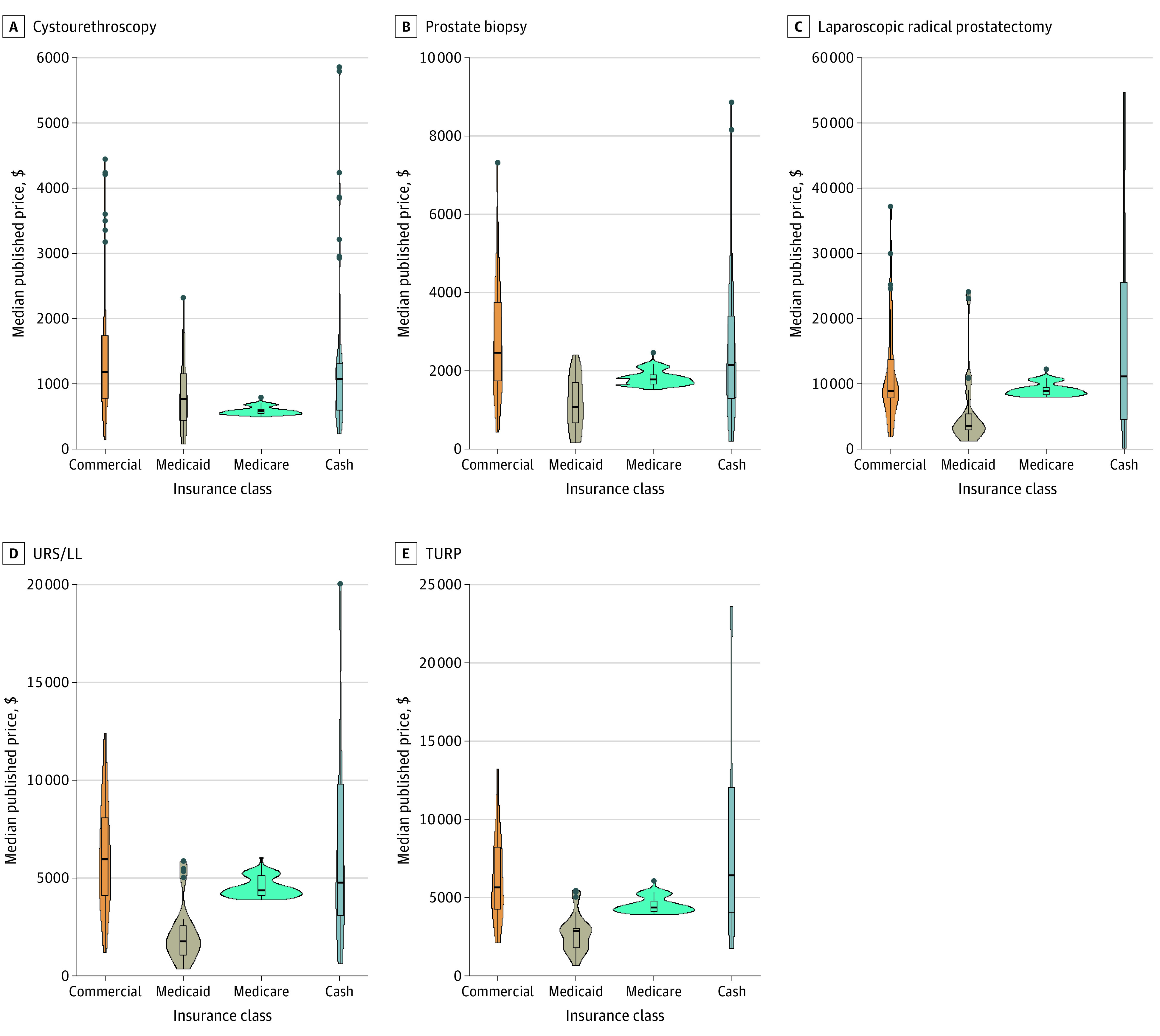
Variations in Procedure Price by Insurance Class TURP indicates transurethral resection of the prostate; URS/LL, ureteroscopy with laser lithotripsy.

**Figure 2.**
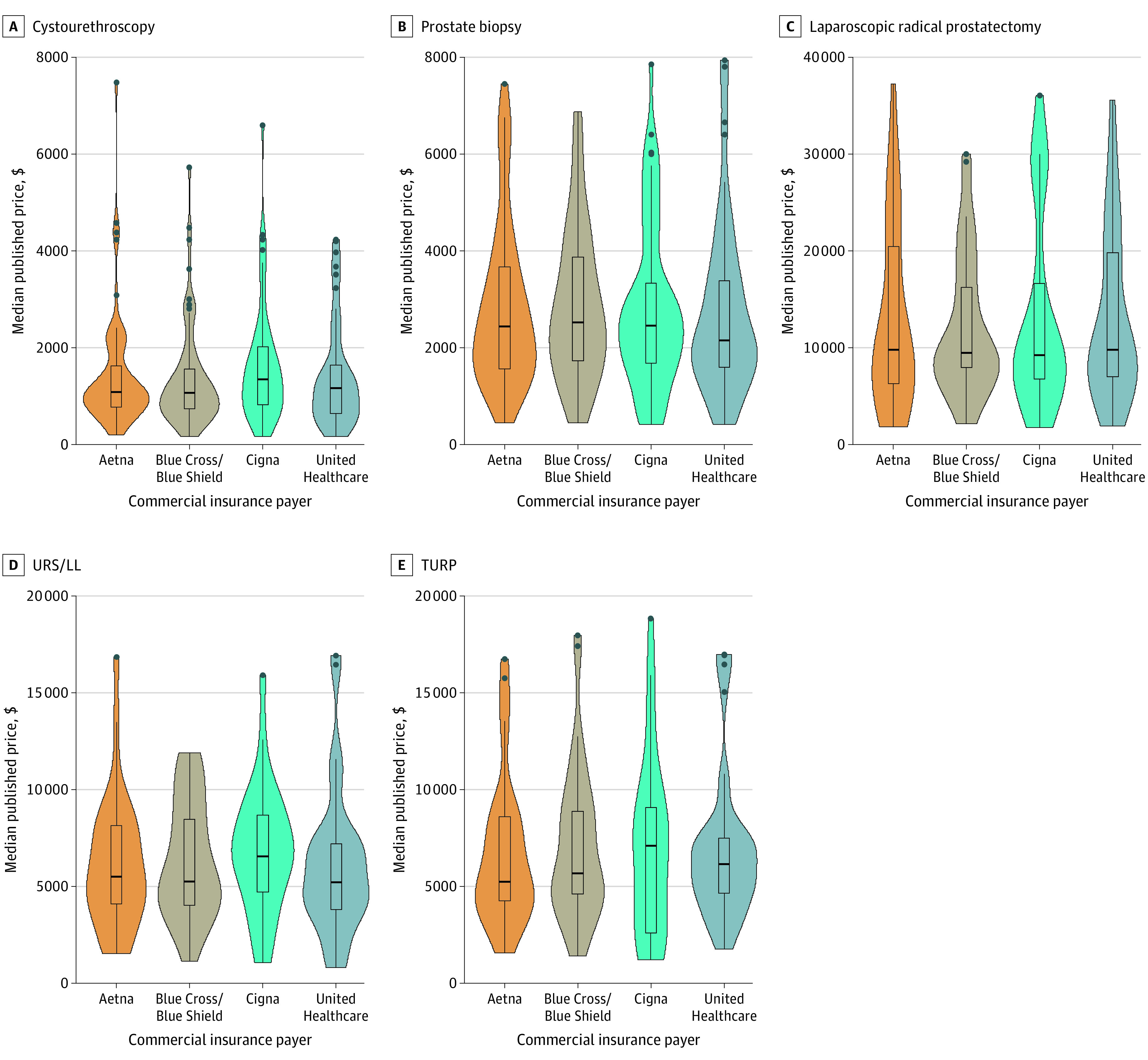
Variations in Procedure Price Among Commercial Insurance Types TURP indicates transurethral resection of the prostate; URS/LL, ureteroscopy with laser lithotripsy.

There were significant variations in negotiated prices among hospitals (Table 3). There were significant variations in the prices of cystoscopy (χ^2^_3_ = 85.9; *P* = .001), prostate biopsy (χ^2^_3_ = 64.6; *P* = .001), prostatectomy (χ^2^_3_ = 24.4; *P* = .001), TURP (χ^2^_3_ = 51.3; *P* = .001), and URS/LL (χ^2^_3_ = 63.0; *P* = .001) by insurance type. There was a significant difference in price among the 4 insurance classes for all 5 procedures (Figure 1). The prices ranged from $572 to $1179 for cystouretheroscopy, $1081 to $2465 for prostate biopsy, $3559 to $11 044 for radical prostatectomy, $2894 to $6445 for TURP, and $1746 to $5962 for URS/LL. Among the 4 private insurers, prices ranged from $1044 to $1316 for cystouretheroscopy, $2155 to $2514 for prostate biopsy, $9186 to $9800 for radical prostatectomy, $5228 to $7098 for TURP, and $5250 to $6588 for URS/LL.There were no significant differences in price among the different commercial insurance types (Figure 2). Results from the sensitivity analyses, during which we compared both the lowest (eTable 1 in Supplement 1) and highest (eTable 2 in Supplement 1) negotiated prices among insurance classes and among commercial insurance types, were similar.

**Table 3.** Cash Fee, Medicaid, Medicare, and Private Insurance Prices for Cystourethroscopy, Prostate Biopsy, Prostatectomy, Transurethral Resection of the Prostate, and Ureteroscopy With Laser Lithotripsy

Procedure and insurance type (*CPT* code)	Price, median (IQR), $
Cystourethroscopy (52000)	
Commercial	1179 (766-1728)
Medicaid	757 (440-1147)
Medicare	572 (534-606)
Cash	1073 (591-1308)
Prostate biopsy (55700)	
Commercial	2465 (1740-3747)
Medicaid	1081 (667-1711)
Medicare	1784 (1667-1890)
Cash	2149 (1284-3392)
Prostatectomy (55866)	
Commercial	8894 (7838-13 631)
Medicaid	3559 (2882-5353)
Medicare	8891 (8306-9391)
Cash	11 044 (4434-25 555)
Transurethral resection of the prostate (52601)	
Commercial	5666 (4264-8209)
Medicaid	2894 (1770-3017)
Medicare	4392 (4116-4771)
Cash	6445 (4034-12 054)
Ureteroscopy with laser lithotripsy (52353)	
Commercial	5962 (4103-8084)
Medicaid	1746 (1055-2547)
Medicare	4392 (4100-5122)
Cash	4784 (3085-9812)

Abbreviation: *CPT*, *Current Procedural Terminology*.

## Discussion

This cross-sectional analysis of 153 academic hospitals found that compliance with the Price Transparency Final Rule remains below 30% and that there are large variations in the price of procedures among hospitals. There were also significant differences in price among the different insurance classes for all 5 common urologic procedures. Most strikingly, the cash price was lower than the Medicare, Medicaid, and commercial price at 16% of hospitals. In other words, in certain situations, health care costs would be reduced if patients omitted their insurance information and paid out of pocket. For all 5 procedures, there were no significant differences in prices among the 4 commercial insurance types.

The persistently large variations in the price of urologic procedures, among academic medical centers and by insurance class, reveal that the Price Transparency Final Rule has not had its intended effect. One reason the Final Rule has fallen short is that compliance with the legislation is low. We found that only 29% of academic hospitals are compliant. These compliance rates are similar to those that have been previously reported, although 1 small study,^12^ which only included US News Top 21 Hospitals, found higher rates of price reporting.^12,15,18^ Among hospitals that are compliant, we found that prices are reported more often for lower cost procedures, and similar findings have been reported previously.^15^ The higher rates of price reporting for lower cost procedures, despite possible fines of up to $300 per day for noncompliance,^7^ suggests that some hospitals are purposefully omitting cost information. This implies that either fines are not being imposed or are not sufficiently costly to hospitals. We suspect that the primary issue is the former, because as of June 9, 2022, of the 5239 total hospitals registered with CMS, only 2 hospitals have been fined for noncompliance.^19,20^ To improve compliance with price transparency, fines actually need to be enforced and possibly increased.

Even if there were stricter enforcement and higher rates of compliance, cost differentials would be eliminated only by the competitive pressures of price shopping. Previous research^21,22^ has shown that even when pricing information is available, patients often do not shop for medical goods or services. Determining hospitals’ costs can be difficult and time intensive. For example, 1 study^23^ found that only 77% of academic hospitals’ websites presented consumer-friendly (not just machine readable) pricing information. Among these, 18% had usability issues, including long search times.^23^ Patients will price shop only if the associated cost savings are greater than the associated labor costs. For price transparency to be effective, new health care policy should address reducing this patient burden.

### Limitations

Our findings should be interpreted in the context of several limitations. Most important are the limitations associated with the data and data reporting. In its current form, price transparency legislation only partially lifts the shroud of mystery surrounding patient billing. For example, the legislation applies to hospitals only, even though hospital care accounts for only approximately 30% of health care expenditures.^24^ Moreover, the costs hospitals disclose may be incomplete because they are not required to include the cost of independent practitioners, who often deliver hospital-based care and constitute a sizable portion of total care costs.^25^

Ambiguities in the legislation allow hospitals to omit potentially important pricing information without being obviously noncomplaint. One example is that hospitals must list both the inpatient and outpatient prices for services only if these prices are different. If only 1 price is listed, the patient cannot be sure whether prices are the same or whether the hospital is noncomplaint and is listing only the lower price. A similar equivocation requires hospitals to list the gross charge and the discounted cash price, but CMS notes that if hospitals have not yet “determined a discounted cash price for self-pay consumers…the hospitals’ cash price would simply be the gross charges.”^7^ As written, hospitals can either not offer a discounted cash price and instead use the gross charge, which is marked up to maximize hospital revenue, or conceal information from patients about lower prices and can technically appear compliant.^26^

Another limitation is the potential lack of generalizability to nonacademic centers and other specialties. A previous study^17^ of the hospital factors associated with price transparency found that teaching hospitals were more compliant in 1 of their 4 models. In addition, although payers with larger enrollment should theoretically be able to achieve better pricing, because we did not have information about insurer-level enrollment, we were unable to account for the impact of enrollment size on prices. It is also likely that there are inaccuracies associated with Turquoise Health’s automatic data compilation and that we are overestimating hospital compliance. The data were sourced from machine-readable files, and we did not assess whether hospitals also provided consumer-friendly pricing tools, both of which are required by the mandate. Despite these limitations, our findings reveal an opportunity to reduce health care spending as well as elucidate how future health care policies could improve price transparency.

## Conclusions

More than 1 year after the implementation of the Price Transparency Final Rule, this cross-sectional study found that there are still large variations in the prices of urologic procedures among academic hospitals and by insurance class. To improve efficacy, CMS must enforce penalties for noncompliance, and new policies should emphasize easy accesses to pricing information.

## Notes

Supplement 1. **eTable 1.** Median of the Minimum Prices and Interquartile Range (IQR) of the Minimum Prices for the Commercial Price, Medicaid Price, Medicare Reference Price, and Cash Price for Cystourethroscopy, Prostate Biopsy, Prostatectomy, Transurethral Resection of the Prostate (TURP), and Ureteroscopy With Laser Lithotripsy (URS/LL) **eTable 2.** Median of the Maximum Prices and Interquartile Range (IQR) of the Maximum Prices for the Commercial Price, Medicaid Price, Medicare Reference Price, and Cash Price for Cystourethroscopy, Prostate Biopsy, Prostatectomy, Transurethral Resection of the Prostate (TURP), and Ureteroscopy With Laser Lithotripsy (URS/LL)

Supplement 2.
Data Sharing Statement
