# Supplementary material for: Large Variations in the Prices of Urologic Procedures at Academic Medical Centers 1 Year After Implementation of the Price Transparency Final Rule
Source: JAMA Netw Open. 2023 Jan 5;6(1):e2249581. doi: 10.1001/jamanetworkopen.2022.49581 (PMC9857154; doi:10.1001/jamanetworkopen.2022.49581)
Supplement: Supplement 1. — eTable 1. Median of the Minimum Prices and Interquartile Range (IQR) of the Minimum Prices for the Commercial Price, Medicaid Price, Medicare Reference Price, and Cash Price for Cystourethroscopy, Prostate Biopsy, Prostatectomy, Transurethral Resection of the Prostate (TURP), and Ureteroscopy With Laser Lithotripsy (URS/LL) eTable 2. Median of the Maximum Prices and Interquartile Range (IQR) of the Maximum Prices for the Commercial Price, Medicaid Price, Medicare Reference Price, and Cash Price for Cystourethroscopy, Prostate Biopsy, Prostatectomy, Transurethral Resection of the Prostate (TURP), and Ureteroscopy With Laser Lithotripsy (URS/LL) [file jamanetwopen-e2249581-s001.pdf]

Supplemental Online Content

Gul ZG, Sharbaugh DR, Guercio CJ, et al. Large variations in the prices of urologic procedures at academic medical centers 1 year after implementation of the Price Transparency Final Rule. *JAMA Netw Open*. 2023;6(1):e2249581. doi:10.1001/jamanetworkopen.2022.49581

eTable 1. Median of the Minimum Prices and Interquartile Range (IQR) of the Minimum Prices for the Commercial Price, Medicaid Price, Medicare Reference Price, and Cash Price for Cystourethroscopy, Prostate Biopsy, Prostatectomy, Transurethral Resection of the Prostate (TURP), and Ureteroscopy With Laser Lithotripsy (URS/LL)

eTable 2. Median of the Maximum Prices and Interquartile Range (IQR) of the Maximum Prices for the Commercial Price, Medicaid Price, Medicare Reference Price, and Cash Price for Cystourethroscopy, Prostate Biopsy, Prostatectomy, Transurethral Resection of the Prostate (TURP), and Ureteroscopy With Laser Lithotripsy (URS/LL)

This supplemental material has been provided by the authors to give readers additional information about their work.

© 2023 Gul ZG et al. *JAMA Network Open.*

eTable 1. Median of the Minimum Prices and Interquartile Range (IQR) of the Minimum Prices for the Commercial Price, Medicaid Price, Medicare Reference Price, and Cash Price for Cystourethroscopy, Prostate Biopsy, Prostatectomy, Transurethral Resection of the Prostate (TURP), and Ureteroscopy With Laser Lithotripsy (URS/LL)

Cystourethroscopy (52000)		
	Median of the Min Prices ($)	IQR (25th-75th percentile, $)
Commercial	630	444-1058
Medicaid	494	235-969
Medicare	572	534-606
Cash	1073	591-1308
Prostate Biopsy (55700)		
	Median of the Min Prices ($)	IQR (25th-75th percentile, $)
Commercial	1706	1156-2795
Medicaid	838	392-1222
Medicare	1784	1667-1890
Cash	1966	1196-3392
Prostatectomy (55866)		
	Median of the Min Prices ($)	IQR (25th-75th percentile, $)
Commercial	7838	4187-9800
Medicaid	3297	2180-5300
Medicare	8891	8306-9391
Cash	11044	4434-25555
TURP (52601)		
	Median of the Min Prices ($)	IQR (25th-75th percentile, $)
Commercial	4187	2638-5076
Medicaid	2534	1630-2991
Medicare	4392	4116-4771
Cash	6445	4034-12054
URS/LL (52353)		
	Median of the Min Prices ($)	IQR (25th-75th percentile, $)
Commercial	3887	2322-4808
Medicaid	1658	1055-2109
Medicare	4391	4100-5122
Cash	4784	3085-9812

© 2023 Gul ZG et al. *JAMA Network Open.*

eTable 2. Median of the Maximum Prices and Interquartile Range (IQR) of the Maximum Prices for the Commercial Price, Medicaid Price, Medicare Reference Price, and Cash Price for Cystourethroscopy, Prostate Biopsy, Prostatectomy, Transurethral Resection of the Prostate (TURP), and Ureteroscopy With Laser Lithotripsy (URS/LL)

Cystourethroscopy (52000)		
	Median of the Max Prices ($)	IQR (25th-75th percentile, $)
Commercial	1792	1064-3074
Medicaid	957	612-1614
Medicare	572	534-606
Cash	1073	607-1308
Prostate Biopsy (55700)		
	Median of the Max Prices ($)	IQR (25th-75th percentile, $)
Commercial	3315	2441-4742
Medicaid	1461	904-1917
Medicare	1784	1667-1890
Cash	2149	1324-3471
Prostatectomy (55866)		
	Median of the Max Prices ($)	IQR (25th-75th percentile, $)
Commercial	13970	9787-29035
Medicaid	3637	3439-8007
Medicare	8891	8306-9391
Cash	11044	4434-25555
TURP (52601)		
	Median of the Max Prices ($)	IQR (25th-75th percentile, $)
Commercial	8586	6872-11759
Medicaid	2991	2066-3725
Medicare	4392	4116-4771
Cash	6445	4034-12054
URS/LL (52353)		
	Median of the Max Prices ($)	IQR (25th-75th percentile, $)
Commercial	8326	6293-9993
Medicaid	1907	1449-2624
Medicare	4391	4100-5122
Cash	4784	3085-9812

© 2023 Gul ZG et al. *JAMA Network Open.*
