# Supplementary material for: Large Variations in the Prices of Urologic Procedures at Academic Medical Centers 1 Year After Implementation of the Price Transparency Final Rule
Source: JAMA Netw Open. 2023 Jan 5;6(1):e2249581. doi: 10.1001/jamanetworkopen.2022.49581 (PMC9857154; doi:10.1001/jamanetworkopen.2022.49581)
Supplement: Supplement 2. — Data Sharing Statement [file jamanetwopen-e2249581-s002.pdf]

Data Sharing Statement

Gul. Large Variations in the Prices of Urologic Procedures at Academic Medical Centers 1 Year After Implementation of the Price Transparency Final Rule. *JAMA Netw Open*. Published January 05, 2023. doi:10.1001/jamanetworkopen.2022.49581

Data
Data available: No
